# Meta-analysis identifies the effect of dietary multi-enzyme supplementation on gut health of pigs

**DOI:** 10.1038/s41598-021-86648-7

**Published:** 2021-03-31

**Authors:** Sivasubramanian Ramani, Neeraja Recharla, Okhwa Hwang, Jinyoung Jeong, Sungkwon Park

**Affiliations:** 1 Department of Food Science and Biotechnology, Sejong University, 209 Neungdong-ro, Seoul, 05006 Korea; 2 National Institute of Animal Science, RDA, Jeonju, 55365 Jeollabukdo Korea

**Keywords:** Applied microbiology, Microbial communities, Microbiology

## Abstract

Gut health though is not well defined the role of gastrointestinal tract is vital if an animal must perform well. Apart from digestion, secretion, and absorption gut is harbored with consortium of microbiota which plays a key role in one’s health. Enzymes, one of the alternatives for antibiotics with beneficial effects on digestion and consistency of food and its effect on gut health. The effect of enzyme supplementation on gut health is not well established and the objective of this meta-analysis is to investigate if the enzyme supplement has influence on gut. This meta-analysis includes 1221 experiments which has single enzyme studies and or studies with multiple enzyme complexes but not challenged. The ratio of *Lactobacillus* and *E. coli* is related to ADFI which showed comparatively lower negative correlation coefficient, with − 0.052 and − 0.035, respectively, whose *I*^2^ values are below 25%, showing that these studies show a significantly lower level of heterogeneity. Correlation between villus height, crypt depth, their ratio and fatty acid is also assessed, and it showed that when the animal is supplemented with two enzyme complexes resulted in positive gut health rather than the single or more than two enzymes.

## Introduction

The gastrointestinal tract (GIT) is the interface at which digestion, secretion, and absorption occurs. In, pigs, the digestive system is monogastric^1^. The pig’s primitive digestive system is formed when it is at twelve days of gestation period from the entoderm, however, the maturity of the GIT develops during the pig’s perinatal life. Most of the digestive capabilities are observed during the perinatal stage^2–6^. Hitherto, there is no known definition for the health of the gut, or gut health. According to the World Health Organization, gut health is broadly defined as follows: (1) effective digestibility and absorption of food, (2) absence of gut illness, (3) normal and stable gut microbiota, (4) good immune status, and (5) a status of well-being^7^.

The focus of global livestock industries is the use of economical, efficient, and sustainable production methods without compromising the quality or quantity of the products, as this determines the potential of obtaining good economic returns. Globally, swine is the most highly consumed meat and also serves as good animal model. Despite many milestones having been achieved in research, there is still room to improve the efficiency of the production of swine^8,9^. An efficient production using swine is obtained by maintaining the swine from the piglet stage to the slaughter stage with good health and good feed concomitantly without disturbing the animal’s welfare or environment^10^. During this process, pigs consume several types of feed and are subjected to various feeding strategies. Moreover, for economic reasons, many alternative feeds have been used that are difficult for the digestion (example, non-digestible fiber rich feed) in a monogastric animal. Furthermore, the use of antibiotics in animal feed as a growth promotor should be restricted due to the possibility of arising antibiotic resistance and environmental issues.

The use of enzymes in feed supplement for promoting gut health in pigs is expected to increase as an alternative to antibiotics and a solution to economic concerns. Enzymes can also be used very effectively as an alternative to probiotics or essential oils^11,12^. Due to this increasing interest, this study is conducted to meet the needs of long-term. Yet, no-good studies are made to investigate the efficacy of enzymes in feed supplement in terms of the gut health of pigs or any livestock animal.

Meta-analysis uses a statistical technique in a systematic review to derive and integrate the results from all the included studies^13,14^. The effect size indices help to perform the quantification of all the collected study results using the same metrics. The assessment of heterogeneity is quantified by Cochran *Q* statistic, *I*^2^, *H*^2^, and^15^ *τ*^2^. Density plotting is a good approach for visualizing data in order to confirm whether the data is normally distributed or whether there is any skew or kurtosis.

## Results and discussion

**Figure 1:**
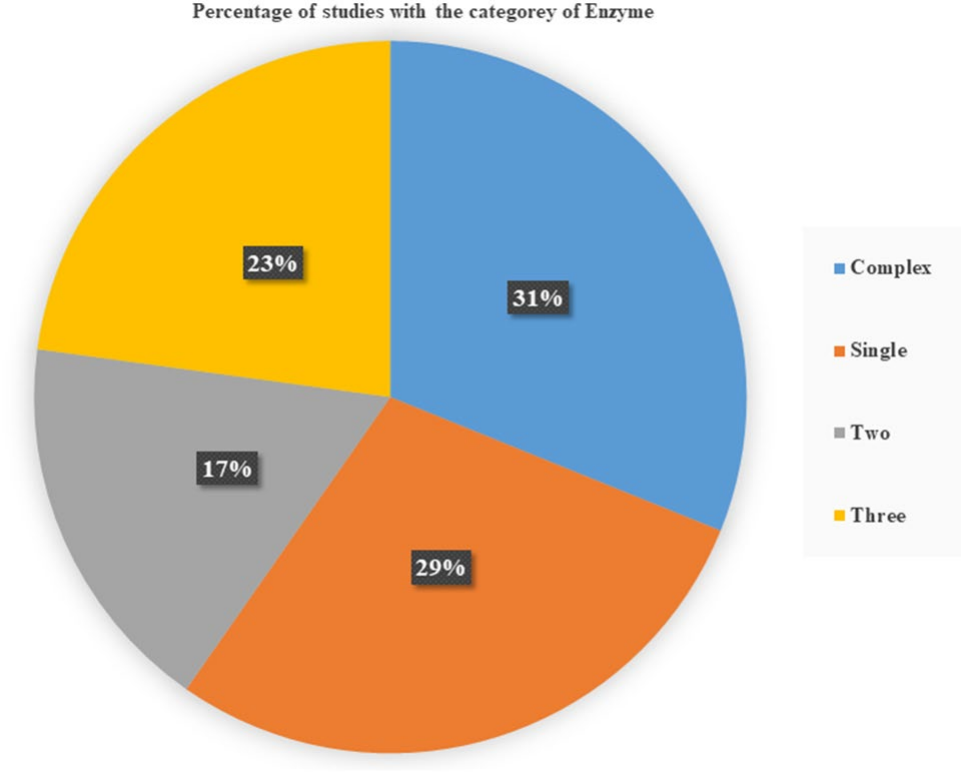
Pie-chart showing the total percentage of the studies by each enzyme supplement category.

The titles and abstracts of 112 articles are screened before selecting 17 articles (Supplementary 1) for consideration in this study. Overall, 1221 experimental treatments were considered for this study after an elaborate search and duplicates were excluded. The feed system categories are ad libitum, controlled, and restricted feed which are used in 1038, 157, and 26 treatments, respectively. Most of the animal studies involved ad libitum feeding systems. The treatments selected here has at least one type of enzyme supplements, ranging from single enzyme supplements to studies with multiple enzyme complexes. There are 349 treatments in which one type of enzyme is used, 213 treatments in which two types of enzyme were supplemented, 279 treatments in which three types of enzymes were supplemented, and 380 treatments in which more than three types of enzyme were supplemented, categorized as multi-enzyme complex. Table 1 shows the characteristics of the feeding methods with the type of enzyme supplement. The total percentage of the studies for each enzyme category is shown in Fig. 1, as follows: multi-enzyme complex studies made up 31% of the database, single enzyme supplementation 29%, two-enzyme supplementation 17%, and three-enzyme supplementation 23%.

**Table 1:** Enzyme supplement category based on feeding method.

Feed type	Complex	Single	Three	Two	Grand total
Ad libitum	380	311	279	68	1038
Controlled		12		145	157
Restricted feed		26			26
Grand total	380	349	279	213	1221

The selected articles are reported with the different enzyme supplementation which are listed in the Table 2. The supplemented enzymes are categorized into carbohydrase, protease, phytase and lipase. Though many studies included single type of enzyme which are carbohydrase, protease and lipase the individual enzymes are little varied based on the choice of feed, herein carbohydrase are α-Galactosidase and xylanase supplemented as single enzymes, and xylanase was always added in combination with other carbohydrase is β-Glucanase in case of single type of enzyme supplement. Apart from this, some treatments included protease and lipase as individual enzyme supplement. In combination supplemented articles with carbohydrase and protease, xylanase is present in most of the treatments. In case of two types of enzyme supplements, it is 3 different enzymes are feed where 2 enzymes are carbohydrase which are xylanase and amylase. The other type added is protease which is extracted from bacterial source. Phytase is feed in two or three enzyme type combination only.

**Table 2:** Types of enzymes as feed supplement utilized in the meta-analysis.

S. no	Study	Total enzymes	Total enzyme types	Enzyme type	Enzymes
1	Pan, B. et al.,	1	1	Carbohydrase	α-Galactosidase
2	Lan, R. et al.,	1	1	Carbohydrase	Xylanase
3	Passos, A. A. et al.,	1	1	Carbohydrase	Xylanase
4	Zuo, J. et al.,	1	1	Protease	Protease
5	Tactacan, G. B. et al.,	1	1	Protease	Protease
6	Zhang, S. et al.,	1	1	Lipase	Lipase
7	Owusu-Asiedu, A. et al.,	2	1	Carbohydrase	Xylanase and β-Glucanase
8	O’Connell, J. M. et al.,	2	1	Carbohydrase	Xylanase and β-Glucanase
9	Jiang, X. R. et al.,	2	1	Carbohydrase	Xylanase and β-Glucanase
10	Chen, Q. et al.,	2	1	Carbohydrase	Bacterial Xylanase and Fungal Xylanase
11	Li, Y. et al.,	3	2	Carbohydrase and Protease	Amylase, Protease and Xylanase
12	Yi, J. Q. et al.,	3	2	Carbohydrase and Protease	Amylase, Protease and Xylanase
13	Zhang, G. G. et al.,	3	2	Carbohydrase and Protease	Amylase, Protease and Xylanase
14	Jo, J. K. et al.,	3	2	Carbohydrase and Protease	Amylase, Mannanase, Protease
15	Kim, J. et al.,	5	3	Carbohydrase, Protease and Phytase	Amylase, Protease, Mannanase, Xylanase, And Phytase
16	Kiarie, E. et al.,	6	1	Carbohydrase	Pectinase, Cellulase, Mannanase, Xylanase, β-Glucanase, Galactanase
17	Wang, Y. et al.,	6	2	Carbohydrase and Phytase	Xylanase, β-Glucanase, Cellulase, Pectinase, Amylase and Phytase

Reference in the table are in the supplementary document.

Most of the experiments are carried using commercial enzymes, accounting for 66.83% of the treatments, followed by enzymes of bacterial and fungal origin, with 23.58%. These two types of enzymes account for 90.41% of the database created. On the other hand, treatments where the enzymes of bacterial and fungal origin are individually supplemented accounted for with 5.24% and 5.48%, respectively, along with other types of feed administered to the pigs in the different studies, as presented in Table 3.

**Table 3:** Consolidated table with feed type and method, enzyme type and its origin.

Count of feed type	Ad libitum	Controlled	Restricted feed	Grand total
**Complex**	**380**			**380**
**Bacteria and Fungi**	**148**			**148**
Corn and SBM	148			148
**Commercial**	**232**			**232**
Corn and SBM	57			57
Corn and SBM with EO	57			57
Flaxseed and carbohydrase	59			59
No flaxseed and carbohydrase	59			59
**Single**	**311**	**12**	**26**	**349**
**Bacteria**	**59**	**5**		**64**
Corn and SBM		5		5
Fish-soybean meal	33			33
Soybean based diet	26			26
**Commercial**	**252**		**26**	**278**
2.5% soybean oil—Corn and SBM	36			36
5% soybean oil—Corn and SBM	36			36
Corn and SBM	18		26	44
Soybean meal	162			162
**Fungi**		**7**		**7**
Corn and SBM		7		7
**Three**	**279**			**279**
**Bacteria and Fungi**	**122**			**122**
Corn and SBM	122			122
**Commercial**	**157**			**157**
Corn and SBM	107			107
Stachyose-corn and SBM	50			50
**Two**	**68**	**145**		**213**
**Bacteria and Fungi**	**18**			**18**
Mixed grain-based diets	18			18
**Commercial**	**50**	**85**		**135**
Barley wheat-based diets		45		45
Corn and SBM	41			41
EO and Barley wheat-based diets		40		40
Wheat and barley	9			9
**Fungi**		**60**		**60**
Barley based diet		36		36
Wheat based diet		24		24
Grand total	1038	157	26	1221

Flow of information through the stages of the meta-analysis.

### Effects of enzymes in gut health

An in-depth meta-analysis of digestion, a gut health response variable processed with subclasses average daily feed intake (ADFI), average daily gain (ADG), crude protein (CP), gross energy (GE), gain to feed ratio (G:F) and the *Lactobacillus* : *E. coli* (La:Ec) ratio, is conducted. In the case of ADFI, the estimates showed a significantly lower heterogeneity with a total heterogeneity and a standard error (SE) of 0.0055 and 0.0043, respectively, whose total heterogeneity by total variability, *I*^2^, is 18.81%. The heterogeneity of *Q*-statistic is 69.0841, with a p-value of 0.4068 (Table 4). The model estimate is − 0.0525 with a standard error (SE) of 0.0242, whose z-score is − 2.1726 and a 95% class interval (CI) upper bound and lower bound of − 0.0051 and − 0.0998, respectively. The analysis shows that the studies are not different and hence the results are valid. Overall, the enzyme supplement in the feed, irrelevant on the type and number of enzymes, showed a negative correlation (Table 5). On the other hand, the ADFI and La:Ec subclasses had a lower negative correlation coefficient, with − 0.052 and − 0.035, respectively, and a 95% CI upper bound of − 0.005 and 0.011, respectively, with a lower bound of − 0.099 and − 0.08, whose *I*^2^ values are below 25%, indicating that these studies show a very significant lower level of heterogeneity. Moreover, it is evident that the ratio of *Lactobacillus* and *E. coli* is related to the ADFI. This heterogeneity is dependent on animal age, gender, feed, and other conditions. These results are consistent with other available experimental studies displaying that pigs when fed with enzyme supplemented diet has presented with better gut health even when the pigs are challenged with other pathogens^16^. Hence, the outcomes coherent and indicate that feed intake with specific enzymes plays a critical reduction in pathogens (ex. *E.coli*) or leaky gut, in other words, this can maintain the gut flora with beneficial microbial abundance (ex., *Lactobacillus *sp.) with the static effects towards pathogenic bacteria which is a protective mechanism for maintenance or enhancement of gut health.

**Table 4:** Random-effects model with the *τ*^2^ estimator as restricted maximum-likelihood estimator.

	Test of heterogeneity								Model results					
	*k*	*τ*^2^	se	*τ*	*I*^2^	*H*^2^	*Q-statistic*	*p-*value	Estimate	se	*z-*score	*p-*value	ci.lb	ci.ub
ADFI	68	0.0055	0.0043	0.0739	18.81	1.23	69.0841	0.4068	− 0.0525	0.0242	− 2.1726	0.0298	− 0.0998	− 0.0051 *
ADG	67	0.0406	0.0151	0.2015	63.56	2.74	153.471	< .0001	− 0.2214	0.0397	− 5.5819	< .0001	− 0.2992	− 0.1437 ***
CP	54	0.1029	0.0376	0.3207	69.1	3.24	133.5287	< .0001	− 0.3676	0.0621	− 5.9178	< .0001	− 0.4893	− 0.2458 ***
GE	40	0.1399	0.0548	0.374	73.94	3.84	106.5291	< .0001	− 0.5258	0.0798	− 6.5926	< .0001	− 0.6821	− 0.3695 ***
G:F	68	0.084	0.0284	0.2899	61.31	2.58	150.7349	< .0001	− 0.1753	0.0512	− 3.4271	0.0006	− 0.2756	− 0.0750 ***
La:Ec	53	0.0046	0.0042	0.0681	18.84	1.23	40.9558	0.8652	− 0.0345	0.0231	− 1.4941	0.1351	− 0.0798	0.0108

Significant codes: 0 ‘***’ 0.001 ‘**’ 0.01 ‘*’ 0.05 ‘.’ 0.1 ‘ ’ 1.

**Table 5:** Summary of correlation for Predicted Fisher's r-to-z scores transformed to correlation coefficients.

	Correlation	ci.lb	ci.ub	cr.lb	cr.ub	*τ2*	ci.lb	ci.ub	*τ*	ci.lb	ci.ub	*I2*	ci.lb	ci.ub	*H2*	ci.lb	ci.ub
ADFI	− 0.052	− 0.099	− 0.005	− 0.202	0.1	0.0055	0	0.0143	0.0739	0	0.1195	18.8122	0	37.7129	1.2317	1	1.6055
ADG	− 0.218	− 0.291	− 0.143	− 0.554	0.179	0.0406	0.0459	0.2299	0.2015	0.2143	0.4795	63.5586	66.3639	90.8107	2.7441	2.973	10.8822
CP	− 0.352	− 0.454	− 0.241	− 0.765	0.266	0.1029	0.0491	0.2407	0.3207	0.2215	0.4906	69.0993	51.6135	83.9576	3.2362	2.0667	6.2335
GE	− 0.482	− 0.593	− 0.354	− 0.855	0.22	0.1399	0.0587	0.3085	0.374	0.2423	0.5554	73.9358	54.346	86.2176	3.8367	2.1904	7.2557
G:F	− 0.174	− 0.269	− 0.075	− 0.636	0.381	0.084	0.0431	0.1887	0.2899	0.2076	0.4344	61.3083	44.8259	78.0651	2.5845	1.8124	4.5589
La:Ec	− 0.035	− 0.08	0.011	− 0.174	0.106	0.0046	0	0.0023	0.0681	0	0.0475	18.8399	0	10.1631	1.2321	1	1.1131

### Assessment of publication bias

**Figure 2:**
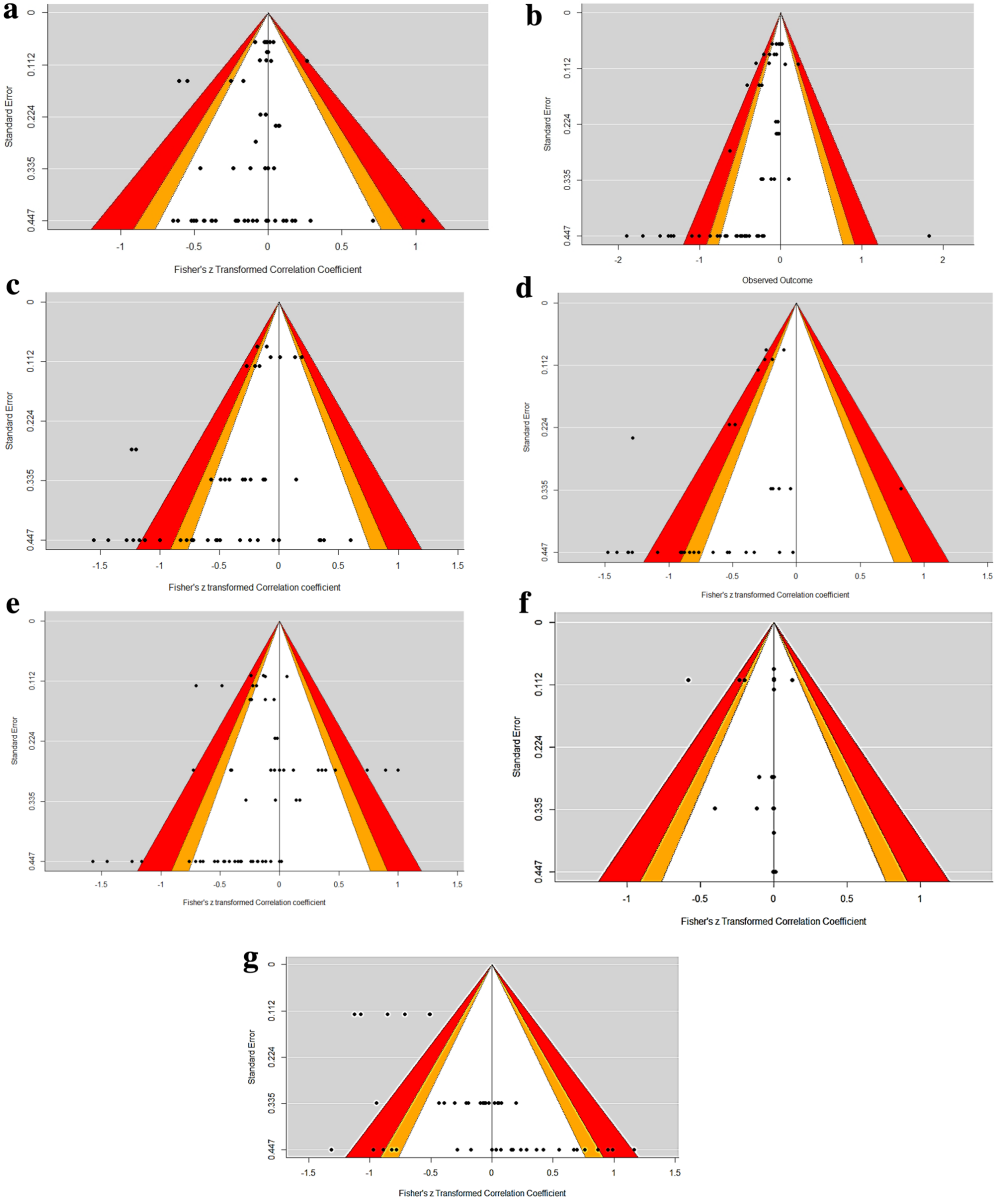
Funnel plots to assess the publication bias. (**a**) ADFI. (**b**) ADG. (**c**) CP. (**d**) GE. (**e**) GF. (**f**) La:Ec. (**g**) Fatty acids. ADFI: Average daily feed intake; ADG: Average daily gain; LB: *Lactobacillus*; Ec: *Escherichia coli*; V/VH: Villus height, C: Crypt depth, CP: Crude protein; GE: Gross energy; GF: Gain to Feed ratio, and La:Ec: *Lactobacillus* : *E. coli* La:Ec ratio.

The publication bias is assessed using funnel plots and further regression tests for funnel plot asymmetry is done in every case (Fig. 2a–g). Using Egger’s mixed effects meta regression model, the publication bias is predicted based on the standard error and rank correlation test for asymmetry, using Kendell’s Tau statistics for ADFI, ADG, CP, GE, G:F, and La:Ec (Table 6). Egger’s test for fatty acids (FA) and La:Ec showed no significance, which indicates that there is no evidence for publication bias in this case. However, although the ADFI and G:F results are non-significant, they are comparatively lower than the former ones. Although, there is no consensus for the beneficiary effects of ADFI, ADG, GE, CP, or G:F in feed supplemented with enzymes, in the case of La:Ec and FA, the effects of enzyme supplementation are observed in some studies^17–19^.

**Table 6:** Tests for publication bias.

	Eggers regression		Rank correlation	
	z	p-value	Kendall’s *τ*	p-value
ADFI	− 2.0393	0.0414	− 0.2204	0.0153
ADG	− 6.1548	< 0.0001	− 0.4452	< 0.0001
CP	− 4.3254	< 0.0001	− 0.2876	0.0064
GE	− 2.8237	0.0047	− 0.4209	0.0006
G:F	− 1.9186	0.055	− 0.2704	0.0031
*La:Ec*	0.0991	0.9211	− 0.6564	< 0.0001
FA	1.2411	0.2146	0.1009	0.3092

### Growth relation with ADFI

**Figure 3:**
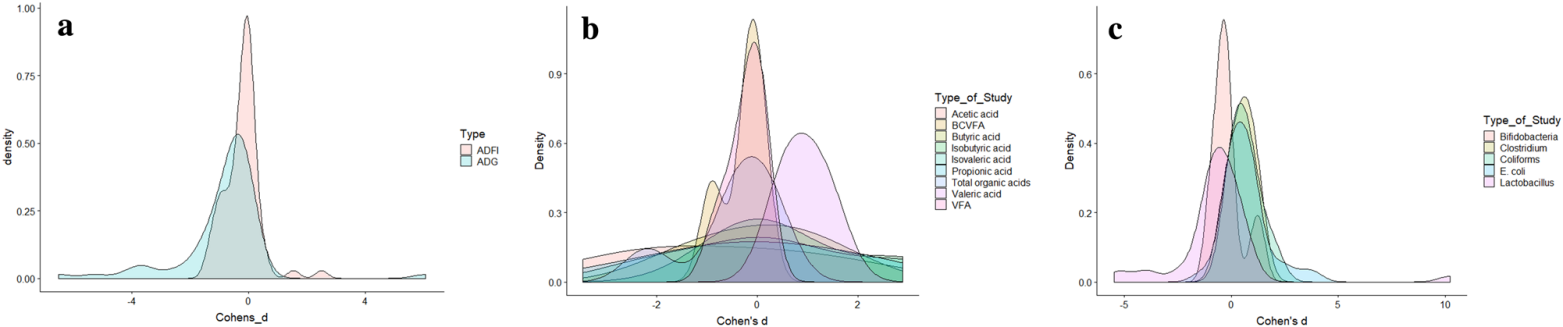
Density plot. (**a**) Density plot of ADFI and ADG data. (**b**) Density plot of fatty acids. (c) Density plot of microbes.

**Figure 4:**
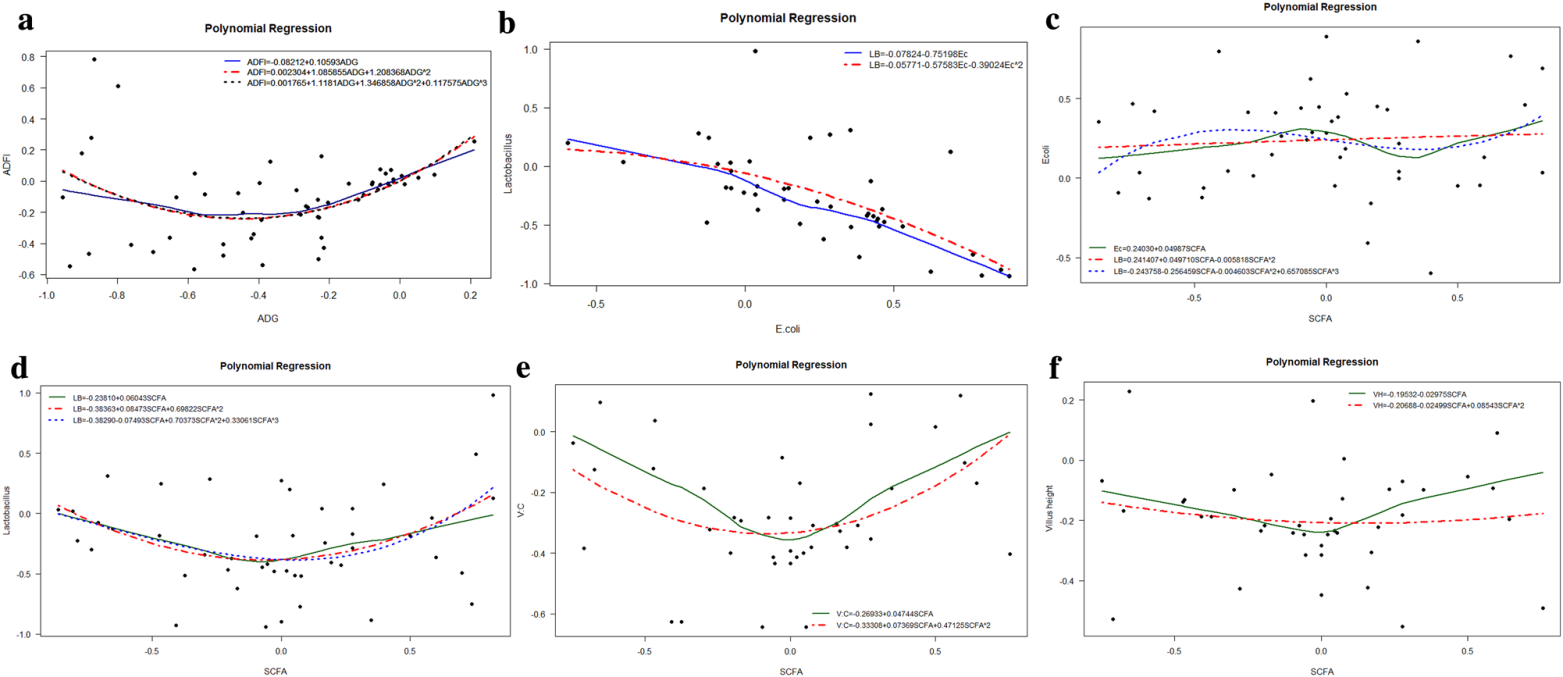
Polynomial regression analysis. (**a**) Polynomial regression analysis of ADFI versus ADG. (**b**) Polynomial regression analysis of Lactobacillus versus *E.coli* (**c**) Polynomial regression analysis of *E.coli* versus fatty acids. (**d**) Polynomial regression analysis of *Lactobacillus* versu Fatty acids. (**e**) Polynomial regression analysis of V:C versus fatty acids. (**f**) Polynomial regression analysis of Villus height versus fatty acids. ADFI: Average daily feed intake; ADG: Average daily gain; LB: *Lactobacillus*; Ec: *Escherichia coli*; V/VH: Villus height, C: Crypt depth, FA/SCFA: fatty acids.

The ameliorating effect of enzyme supplementation on feed appears to be based on the substrate which is added. In enzyme supplementation strategy, as the addition of enzymes is assumed to increase animal performance, in this study we carried out variance–covariance analysis to decipher the enzyme supplement efficacy in animal gut health. Polynomial regression analysis is performed using the ADFI with ADG values. Initially, the data distribution is visualized using density plots (Fig. 3a–c). To fit the model, the *x* variable is cubed when the *y* represented ADFI with an intercept (α) of 0.001 (p = 0.01) and where β_1_ is 1.118, β_2_ is 1.346, and β_3_ is 0.117 (Fig. 4a). This fitted model had a residual standard error (RSE) of 0.2383 and 56 degrees of freedom (DOF), a multiple R-squared (RSD) of 0.1965, an adjusted R-squared (Rad) of 0.1534 with a F-statistic of 4.564 (p-value = 0.006256). Reduction in ADG is ascribed to a reduced ADFI, however in the gut health challenges, growth may be impaired due to an increase in the requirements for the metabolic and digestive processes. Apart from gut health conditions, the G:F ratio also is impaired by other factors like diet composition, age of pigs, other environmental factors also influence. However further studies are needed considering all the factors.

### Influence of enzymes on Lactobacillus versus E.coli

Many subjects have demonstrated that under dysbiosis conditions, the abundance of *E. coli* is higher and the amount of beneficiary bacteria belonging to the genera *Firmicutes* (e.g. *Lactobacillus*) is reduced. *Lactobacillus* is highly associated with gut health factors and animal performance^20^. Thus, the ratio of *Lactobacillus* and *E. coli* has played an important role in the animal’s gut health and overall performance. Here, when *Lactobacillus* is increased, the abundance of *E. coli* is found to decrease. The *α* is − 0.057 when *y* is *Lactobacillus*, with the *x* squared to fit the regression model (Fig. 4b). The estimated coefficients are β_1_ is − 0.575 (p-value = 0.008) and β_2_ is − 0.39024. RSE is 0.309 with 41 DOF. The RSD and Rad are 0.4088 and 0.38, respectively, with an F-statistic of 14.18 (p-value = 0.00002). Thus, it is very clear that the population of *Lactobacillus* negatively regulates the pathogenic *E. coli* population, and the use of enzymes can sometimes enhance the *Lactobacillus* count in the intestine to improve gut health.

Gut health is associated with microbial abundance and diversity as the presence of certain types of microbes are beneficial, since the metabolites produced by these bacteria assist other beneficial bacteria in the microbial network^20–23^. However, the influence of the types or numbers of enzymes on the structure of microbes and fatty acids production in gut has not yet been well established. The changes in substrate availability for microbes is a determining factor in the role of microbes in gut health. In the case of *E. coli*, model fitting did not show any trend or significance regarding fatty acids in the GIT (Fig. 4c). On the other hand, *Lactobacillus* showed a trend and model fitting (Fig. 4d). The intercepts for *E. coli* and *Lactobacillus* are 0.2437 (p-value = 0.000547) and − 0.3829 (p-value = 0.000004), respectively, indicating that when *E. coli* is at zero, the fatty acid intercept is positive, meaning that the production of FA is not dependent on *E. coli.* On the other hand, the *Lactobacillus* intercept is predicted to be a negative value, indicating that if *Lactobacillus* is absent, the production of fatty acid is also absent, which could lead to significant problem. The results are consistent with other published articles where the supplementing the animals with enzyme resulting in reduced pathogens even during challenged conditions and in non-challenged conditions^16,24^.

### Changes in VH:CD ratio

Digested food is absorbed through brush borders, also known as projected villi, and invaginated crypts, which are microanatomical structures that play a crucial role in gut health. These structures are lined with a mucus layer that provides a mucosal barrier. The height of the projected villus and the depth of the crypts are also indicators of GIT health. Many factors influence the villus height and crypt depth ratio (VH:CD), including the type of feed, viscosity, pH, and pathological conditions, among others. Thus, a balance between the physiological and pathophysiological is essential for an adequate VH:CD ratio. The supplementation feed with enzymes may modulate the VH:CD levels, with certain studies reporting that enzyme supplementation increases viscosity, leading to changes in this ratio which result in a leaky bowl^18^. In the case of VH:CD, the intercept coefficient is − 0.33308 with an RSE of 0.1921 and DOF of 37 (Fig. 4e). The RSD is 0.1706, Rad is 0.1257, and the F-statistic is 3.805 (p-value = 0.03). The correlation between VH and fatty acid is predicted to have an intercept of − 0.20688 (p-value = 0.0000007) with an RSE of 0.174 and a DOF of 37 (Fig. 4f). The RSD is 0.01201, Rad is − 0.04139, and the F-statistic is 0.225 (p-value = 0.79).

**Figure 5:**
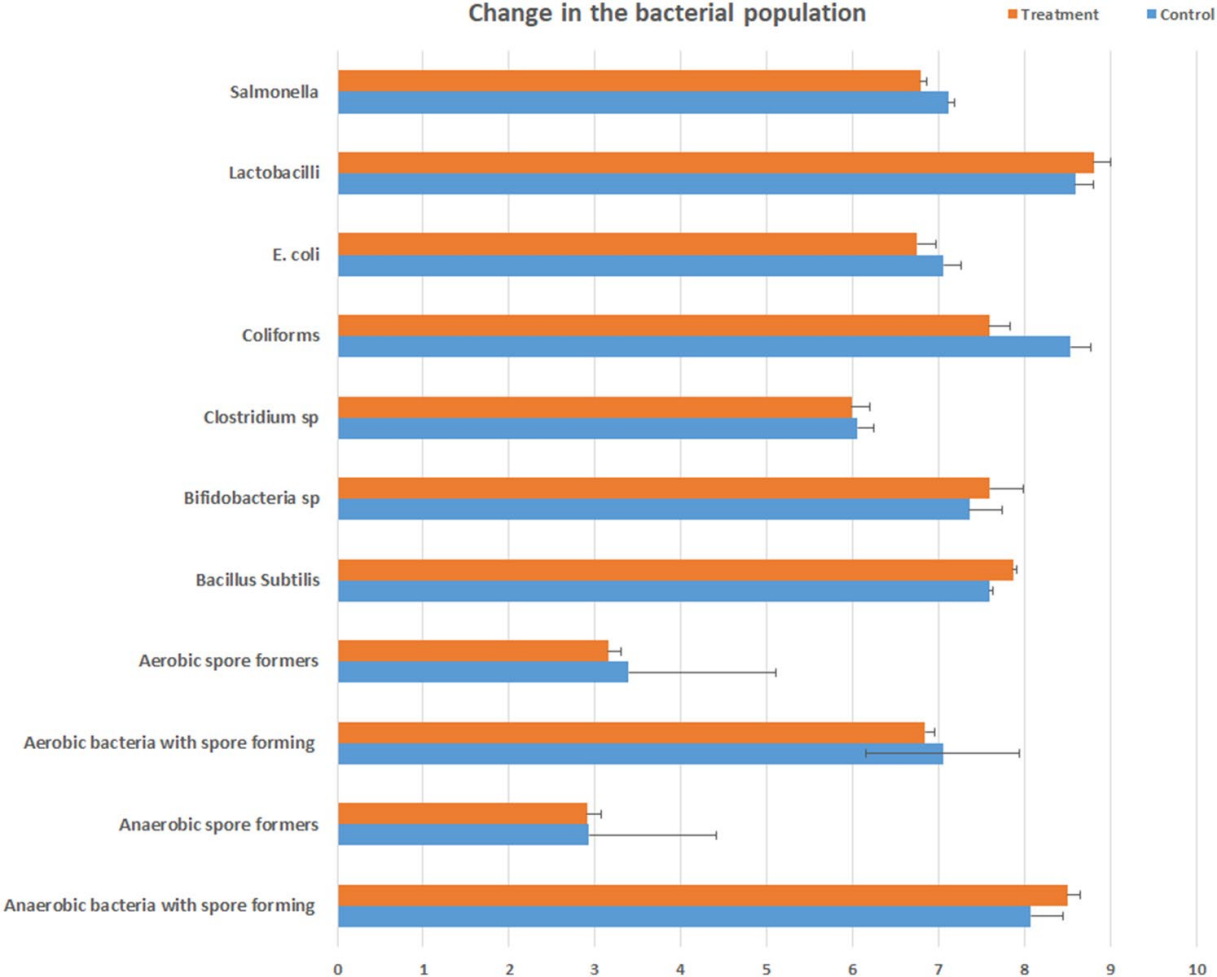
Change in the composition of the gut bacterial population upon enzyme supplement.

**Figure 6:**
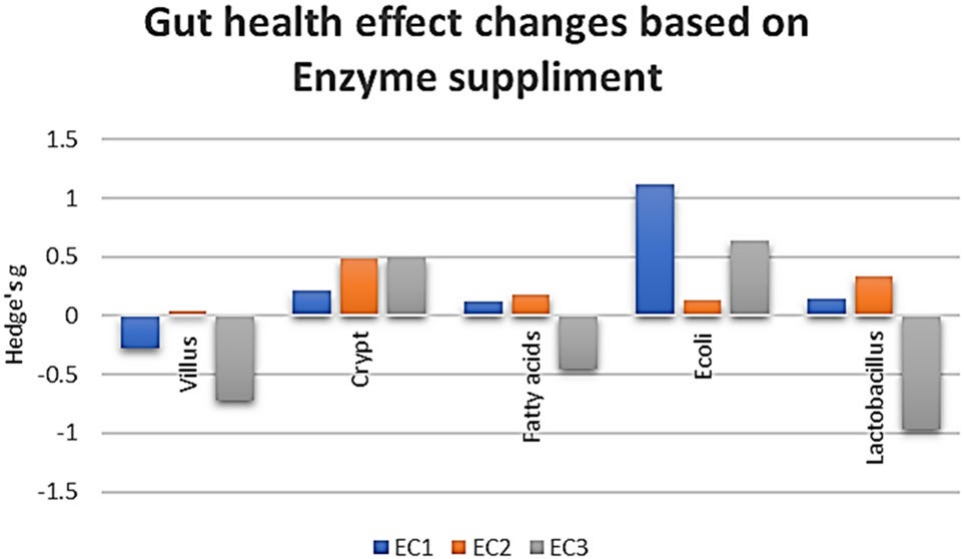
Gut health parameters assessment based on number of enzyme supplement: EC1: single enzyme; EC2: two enzyme complexes; EC3: three or more enzymes.

This study will serve as an important contribution to the body of literature on the impact of enzyme used in animal feed. Here upon compiling the gut microbial structure (Fig. 5), VH, CD, *E. coli*, *Lactobacillus*, and fatty acids against the different types of enzyme complex, and using Hedge’s g for comparison, it is very clear that the usage of two types of enzymes, based on the substrate provided, is more beneficial than the use of a single enzyme or three enzymes and above (Fig. 6). It is also clear that the choice of enzymes based on the substrate is critical in case of animal health. For instance, phytase with carbohydrase supplement improves animal performance by increasing mineral and nutrient absorption. the use of enzymes by animal weight category also plays a role^25^. In case of single enzyme supplement for example xylanase (carbohydrase), animals below 10 kg has showed reduced gut health conditions, where the animals showed reduced V:C ratio mediated by a proinflammatory cytokine tumor necrosing factor alpha (TNFα)^26^. TNFα is well known to trigger the inflammatory responses^27^. Whereas, addition of two enzymes like protease and carbohydrase has benefitted the animals by modifications in digestibility and microbial consortium in contrast^24^. Inflammation in gut is mediated through the upregulation of inflammatory cytokines which tends to leaky gut, where the changes in mucosal barrier function, gut immunity, and microorganisms are observed.

To our knowledge there is no meta-analysis reporting the gut health of pigs based on the enzyme supplement. Also, there is a need for more experiments to be proven regarding the activity of enzymes relation with all the gut health attributes. Based on these results, we propose that use of two enzyme supplement in pig feed programs rather than multiple enzyme supplement which may benefit the pig gut health. However further experiments should be conducted to decipher the correlation between enzyme supplement and all the gut health.

## Methods

### Search strategy

**Figure 7:**
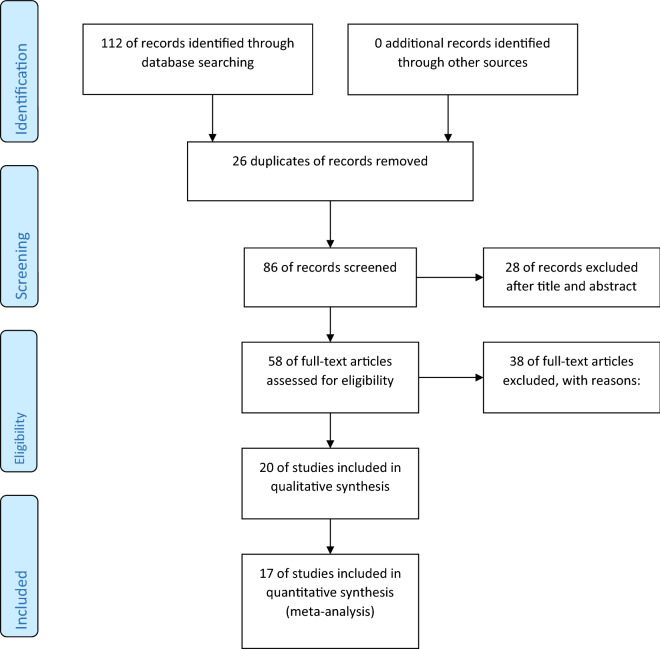
Flow of information through the stages of the meta-analysis.

An intensive computerized literature search is performed to retrieve studies between 2000 and 2018. The search is conducted on PubMed, Web of Science, Google Scholar, and Scopus. The key terms used are “enzymes,” “feed additives,” “feed supplement,” “Swine,” “pig,” “health,” “microorganisms,” and “gut health.” Upon completing the search, all duplicates are removed, and the articles’ titles and abstracts are screened for selection. The full text is then accessed for the knowledge retrieval by two researchers individually. After completing the literature search as per PRISMA guidelines, a flow chart for identification, screening, eligibility, and inclusion is prepared^28^ (Fig. 7).

### Literature inclusion and exclusion criteria

Only articles where swine feed is supplemented with enzymes or enzyme complexes and fed to animals that had never been challenged are considered. The article selection criterion is as follows: (1) a weaning period between 17 and 28 days; (2) a commonly used feed, i.e. soybean meal, corn-soybean meal, barley, or wheat, supplemented ad libitum or via a controlled system; (3) feed is supplemented with at least a single type of enzyme or with a multi-enzyme complex; (4) articles reporting animal performance, gut health parameters (such as SCFA), villus and crypt sizes or ratios, and microorganism studies; (5) articles with mean and SEM are only considered to avoid bias. The exclusion criteria are: (1) studies where animals are challenged with pathogens or toxins; (2) feed supplemented with probiotics or prebiotics.

### Database characteristics

The database is created using Microsoft Access. A form is created to input the data extracted from each article. Each form represented an experimental study from the selected article which fit as a single row in the database. Initially, the database had a total of 1221 rows and 39 columns. Upon including the data from the articles, the effect sizes are computed and added as separate columns. The calculated effect sizes are Cohen’s d, Hedges’ g, Fisher’s z, Cohen’s f, odds ratio, Cox-odds ratio, log odds, Pearson’s r, Cox-log odds, effect size eta square, the standard error of the effect size, the variance of effect size, the lower and upper confidence limits, and the weight factor. As a result, the final database had a total of 55 columns after the addition of the calculated effect sizes for meta-analysis. Every experimental study is encoded with a unique number which is generated automatically when a form is generated freshly, which is used as a general code. Each article is given a sequence number. For inter- and intra-studies, another set of sequence number are allocated, such that a unique code is available for each study retrieved from the selected publications. Thus, the rows in the database represented the treatment and the columns represented the exploratory variables or parameters.

### Data extraction

All the eligible study data are extracted by data entry using the Microsoft Access form, where the publication year of the article, the title of the article, the author names, the sample size, and the mean and SEM are recorded. Moderating variables such as age, weaning period, initial weight, final weight, enzyme type, number of enzymes, enzyme source, enzyme concentration, sample type and time, experiment period, number of animals per pen, genetic background of the animals, feed type, and time are also recorded for analysis. The researchers extracting the data independently cross-verified the data for typographic errors and accuracy.

### Data analysis

The unique codified data is classified based on the following five health response variables: absorption and digestion, gut illness, microbiota, immune status, and status of wellbeing^7^. To determine their effects across multiple studies, as well as within individual studies, the models suggested by^29,30^ are utilized. Dependent and independent variables are defined as per the^29^ approach. R programming version 3.5.1 is used for all data analysis, where the “esc” package is used to calculate the effect size^31^. The calculated effect size is used for the meta-analysis. The packages “metaphor,” “robumeta, “meta,” “metagear,” and/or “mvmeta” are used to conduct the meta-analysis^13,32^. The data is initially explored via graphical analysis to observe the data distribution to check for any heteroscedasticity. Density plots are generated using the Cohen’s d effect size values of the experimental variables.

For conducting a meta-analysis of the effect sizes, the corresponding sample variance are calculated using Cohen’s d and Pearson’s r (r). Here, the r is transformed into Fisher’s z score^33^. The correlation coefficient quantifies for both the direction and strength of the linear relationship between the two quantitative variables and is therefore used often as the resulting measure for meta-analyses. Here, the Fisher's r-to-z transformed correlation coefficient is used for the analysis, as this alternative measure is a bias-corrected version of the previous coefficient^34^. The meta-analysis is carried using the random-effects model for measuring between two quantitative variables. Sequentially, the classified data is explored using graphical analysis with Cohen’s d, effect size, Fisher’s transformed r-to-z, the estimated sample variance, and Hedge’s g. Heterogeneity is estimated using the restricted maximum-likelihood estimator method with random-effect model fitting^35^. The study weights are estimated as inverse-variance, which is a default setting in the “metafor” package.

A quantified heterogeneity is reported based on the *Q*-statistic, *I*^2^, *H*^2^, and *τ*^2^ data. A *Q*-statistic is reported with a DOF with a p-value of the test, which is null hypothesis significance test, which tells us the overall heterogeneity between the studies, but cannot tell us the extent of true heterogeneity^36^. To overcome the limitations of the *Q*-statistic, *τ*^2^ is reported to estimate the total amount of true heterogeneity. In the case of *τ*^2^, it is only dependent on a specific type of effect size estimates, as such, it cannot be used to compare different meta-analyses with different effect sizes. However, the *I*^2^ index can be obtained with a different number of studies and with several types of effect size metrics, which are comparable. *I*^2^ indices of 25%, 50%, and 75% are interpreted as low, moderate, and high, respectively. As shown in previous studies, the between-study variance, *τ*^2^, and the *I*^2^ are directly related^15,37^. The summary of effect size is derived from the estimated model coefficient using the standard error, z-score, p-value, and upper and lower bounds of the confidence interval (CI). For every subgroup meta-analysis is conducted, the results are obtained by further transformation of Fisher's r-to-z scores into correlation coefficients estimates along using the upper and lower bound 95% CI to interpret the results.

### Data visualization

Although a possible heterogeneity is suggested, the data did not point out the specific studies which are influencing this heterogeneity. Thus, Bajaut graphical plotting is carried as a diagnostic plot in an attempt to sort the heterogeneity data. The data used for plotting is the squared Pearson’s residuals of the studies and the influencers, i.e. the standardized squared difference between the fitted value for each study. The data points in the plot that identify the influencing data are the study ID numbers. The most influential studies are observed in the top right quadrant of the plot^38^. Another set of diagnostic plots is also generated and visualized for the potential influencers and outliers using influence plots. Upon computing, we produced a tabular data displaying the difference in the fits, covariance ratios, Cook’s distances, and the diagonal elements of the hat matrices. Any influencing data is marked with an asterisk and the influencers are visualized by different colors in the plot^39,40^.

The point estimates of the study, with the CI and the summary of effect sizes, is plotted on a forest plot to visualize the meta-analysis of the gut health subgroups, or response variables (forest plots are not shown rather the data is presented in table form). The edges of the polygon represent the 95% confidence limit. The publication bias is evaluated based the funnel plots, the Egger’s regression test, and the rank correlation test^41^. Moderator analysis is carried out to estimate the heterogeneity using meta-regression models.

Variance–covariance analysis are performed to determine the effect of enzyme supplementation on the gut health response variables within individual studies and between different studies in which residual analysis is initially conducted. The residual analysis is further graphically verified and observed for normal distribution. The wellbeing of the animals is correlated with performance, hence, the correlation between the coefficients of average daily feed intake is initially regressed against the average daily weight gain to evaluate the biological relevance^29^. Furthermore, to infer the correlation between other gut health factors, polynomial regression model analysis is conducted, as discussed earlier. Factors included comparing *Lactobacillus* to *Escherichia coli*, whose populations indicate the status of the health and dysbiosis condition, since gut health can also be characterized by the villus height (VH), crypt depth (CD), concentration of short chain fatty acids (SCFA), and VH:CD ratios. These parameters are mechanistically inter-related in the support of gut health, hence, we verified their relationship and their associated regulatory mechanisms in the presence of enzyme supplements. The formula for the polynomial regression fit is provided along with the representative plots^30^.

## Supplementary Information

Supplementary Information 1.

Supplementary Information 2.
