# Supplementary material for: Meta-analysis identifies the effect of dietary multi-enzyme supplementation on gut health of pigs
Source: Sci Rep. 2021 Mar 31;11:7299. doi: 10.1038/s41598-021-86648-7 (PMC8012356; doi:10.1038/s41598-021-86648-7)
Supplement: Supplementary file 2 — Supplementary Information 2. [file 41598_2021_86648_MOESM2_ESM.docx]

**Meta-analysis identifies the effect of dietary multi-enzyme supplementation on gut health of pigs**

Sivasubramanian Ramani^1^, Neeraja Recharla^1^, Okhwa Hwang^2^, Jinyoung Jeong^2^, and Sungkwon Park^1*^

1 Department of Food Science and Biotechnology, Sejong University, Seoul 05006, Korea

2 National Institute of Animal Science, RDA, Jeollabukdo 55365, Korea

*Corresponding author: Sungkwon Park (209 Neungdong-ro, Seoul 05006, Korea; Tel: +82-2-3408-2906; email: sungkwonpark@sejong.ac.kr)

ORCID ID:

Sivasubramanian Ramani: 0000-0001-9370-6552

Neeraja Recharla: 0000-0001-9343-7436

Okhwa Hwang: 0000-0003-0681-4577

Jinyoung Jeong: 0000-0002-8670-7036

Sungkwon Park: 0000-0002-7684-9719

Reference:

1. Chen, Q., Li, M. & Wang, X. Enzymology properties of two different xylanases and their impacts on growth performance and intestinal microflora of weaned piglets. *Anim. Nutr.* **2**, 18–23 (2016).

2. Jiang, X. R. *et al.* Effects of a blend of essential oils and an enzyme combination on nutrient digestibility, ileum histology and expression of inflammatory mediators in weaned piglets. *Animal* **9**, 417–426 (2015).

3. Jo, J. K. *et al.* Effects of exogenous enzyme supplementation to corn- and soybean meal-based or complex diets on growth performance, nutrient digestibility, and blood metabolites in growing pigs1. *J. Anim. Sci.* **90**, 3041–3048 (2012).

4. Kiarie, E., Nyachoti, C. M., Slominski, B. A. & Blank, G. Growth performance, gastrointestinal microbial activity, and nutrient digestibility in early-weaned pigs fed diets containing flaxseed and carbohydrase enzyme1,2. *J. Anim. Sci.* **85**, 2982–2993 (2007).

5. Kim, J. *et al.* The microbial pH-stable exogenous multienzyme improved growth performance and intestinal morphology of weaned pigs fed a corn-soybean-based diet. *J. Appl. Anim. Res.* **46**, 559–565 (2018).

6. Lan, R., Li, T. & Kim, I. Effects of xylanase supplementation on growth performance, nutrient digestibility, blood parameters, fecal microbiota, fecal score and fecal noxious gas emission of weaning pigs fed corn-soybean meal-based diet. *Anim. Sci. J.* **88**, 1398–1405 (2017).

7. Li, Y. *et al.* Corn extrusion and enzyme addition improves digestibility of corn/soy based diets by pigs: In vitro and in vivo studies. *Anim. Feed Sci. Technol.* **158**, 146–154 (2010).

8. O’Connell, J. M., Sweeney, T., Callan, J. J. & O’Doherty, J. V. The effect of cereal type and exogenous enzyme supplementation in pig diets on nutrient digestibility, intestinal microflora, volatile fatty acid concentration and manure ammonia emissions from finisher pigs. *Anim. Sci.* **81**, (2006).

9. Owusu-Asiedu, A., Simmins, P. H., Brufau, J., Lizardo, R. & Péron, A. Effect of xylanase and β-glucanase on growth performance and nutrient digestibility in piglets fed wheat–barley-based diets. *Livest. Sci.* **134**, 76–78 (2010).

10. Pan, B., Li, D., Piao, X., Zhang, L. & Guo, L. Effect of dietary supplementation with α-galactosidase preparation and stachyose on growth performance, nutrient digestibility and intestinal bacterial populations of piglets. *Arch. Anim. Nutr. fur Tierernahrung* **56**, 327–337 (2002).

11. Passos, A. A., Park, I., Ferket, P., von Heimendahl, E. & Kim, S. W. Effect of dietary supplementation of xylanase on apparent ileal digestibility of nutrients, viscosity of digesta, and intestinal morphology of growing pigs fed corn and soybean meal based diet. *Anim. Nutr.* **1**, 19–23 (2015).

12. Tactacan, G. B., Cho, S. Y., Cho, J. H. & Kim, I. H. Performance responses, nutrient digestibility, blood characteristics, and measures of gastrointestinal health in weanling pigs fed protease enzyme. *Asian-Australasian J. Anim. Sci.* **29**, 998–1003 (2016).

13. Wang, Y. *et al.* Effect of diet complexity, multi-enzyme complexes, essential oils, and benzoic acid on weanling pigs. *Livest. Sci.* **209**, 32–38 (2018).

14. Yi, J. Q. *et al.* The effects of enzyme complex on performance, intestinal health and nutrient digestibility of weaned pigs. *Asian-Australasian J. Anim. Sci.* **26**, 1181–1188 (2013).

15. Zhang, G. G., Yang, Z. B., Wang, Y., Yang, W. R. & Zhou, H. J. Effects of dietary supplementation of multi-enzyme on growth performance, nutrient digestibility, small intestinal digestive enzyme activities, and large intestinal selected microbiota in weanling pigs. *J. Anim. Sci.* **92**, 2063–2069 (2014).

16. Zhang, S. *et al.* Effect of timing of post-weaning supplementation of soybean oil and exogenous lipase on growth performance, blood biochemical profiles, intestinal morphology and caecal microbial composition in weaning pigs. *Ital. J. Anim. Sci.* **17**, 967–975 (2018).

17. Zuo, J. *et al.* Effect of dietary supplementation with protease on growth performance, nutrient digestibility, intestinal morphology, digestive enzymes and gene expression of weaned piglets. *Anim. Nutr.* **1**, 276–282 (2015).
